# Evaluating a Wearable-Based Pain Monitoring System in Palliative Cancer Care: Usability and Feasibility Study

**DOI:** 10.2196/78098

**Published:** 2026-02-06

**Authors:** Federico Domínguez, Jacqueline Heras, Jhonston Benjumea, Mariana Vallejo, Ericka Parra, Wagner Fiallos, Andrea Villao, Fabricio Pazmiño, Johan Stiens, Bruno da Silva

**Affiliations:** 1 Centro de Tecnologías de Información, Escuela Superior Politécnica del Litoral, Km. 30.5 Vía Perimetral, Guayaquil, Ecuador, 593 42269777; 2 Unidad de cuidados paliativos, SOLCA-Guayaquil, Guayaquil, Ecuador; 3 Universidad Católica de Santiago de Guayaquil, Guayaquil, Ecuador; 4 Department of Electronics and Informatics (ETRO), Vrije Universiteit Brussel, Brussels, Belgium

**Keywords:** palliative care, smart health care, telemedicine, user centered design, wearable devices

## Abstract

**Background:**

Effective pain management is a cornerstone of cancer palliative care, yet it remains challenging in low- and middle-income countries due to limited resources, regulatory constraints, and a lack of objective tools. While wearable technologies offer promise for augmenting pain-related patient-reported outcomes with physiological data, their usability in palliative settings in low- and middle-income countries is underexplored.

**Objective:**

This study aimed to evaluate the technology usability and implementation feasibility of the NEST (Non-intrusive Devices for Telemedicine) system, a low-cost, smartwatch-based pain monitoring solution for palliative cancer care co-designed with health care staff from a cancer hospital in Ecuador.

**Methods:**

An observational usability study was conducted with 7 patients with cancer receiving palliative care treatment, combining hospital- and home-based monitoring phases. We used a qualitative and quantitative approach to assess the usability of the NEST system and to identify sociotechnical factors affecting feasibility using the NASSS (Nonadoption, Abandonment, Scale-up, Spread, and Sustainability) framework.

**Results:**

Quantitative results showed a strong preference for the smartwatch over the mobile phone for submitting patient-reported outcomes (246/296, 83%), with wear-time adherence of the smartwatch ranging from 36% to 92% of the time. Qualitative feedback from patients and health care staff indicated good usability and perceived clinical value, though technical and organizational challenges, such as charging habits, training needs, and dashboard integration into the daily workflow of health care staff, were noted. As for feasibility, most of the complexity was found in the dynamics of the health condition, while the technology shows clear promising signs of having value to patients and health care staff.

**Conclusions:**

Our findings suggest that the commonly reported usability hurdles of a smartwatch-based sociotechnical health solution are surmountable given fluid communication between stakeholders during all stages of design and deployment. The primary threats to feasibility in our context seem to lie in the highly complex and dynamic environment of palliative cancer care, regulatory ambiguity regarding the use of medical devices, and the workload burden on health care staff.

## Introduction

Pain management is a key component of oncology palliative care, as pain remains one of the most common and life-limiting symptoms reported by patients with cancer receiving ambulatory palliative services [1,2]. In low- and middle-income countries (LMICs), precarious health services infrastructure, limited availability of specialized health care personnel, and highly restrictive policies on opioid prescription [3] create additional pressures to implement efficient and effective pain management strategies in outpatient palliative care. Moreover, with global populations aging and the prevalence of oncologic conditions rising, the demand for palliative care is expected to increase significantly in the coming years [4]. In this context, sociotechnical interventions that extend existing telemedicine services have the potential to provide some relief by providing health care staff effective means to monitor a patient’s well-being and optimize their pain management treatment [5-8].

Palliative care is a specialized medical approach focused on improving the quality of life of patients and their families facing life-threatening illnesses through the prevention and relief of distress [9]. In outpatient settings, where care is delivered at home or during scheduled visits, patient-reported outcomes (PROs) typically refer to a patient’s self-assessment of pain and related symptoms, conveyed verbally during scheduled clinical visits [6,10] or recorded manually using paper-based questionnaires by the patient or their caregiver. PROs are considered the gold standard for pain assessment and management in this context [11-13]. However, they are inherently subjective, influenced by cultural, contextual, and psychosocial factors [12-14], and susceptible to issues, such as recall bias and patient noncompliance [15].

Sensor-enabled technologies, including wearable devices and smartphones, have increasingly been adopted to collect real-time digital biomarkers and PROs in outpatient settings across various clinical conditions [6,10,14]. Recent studies suggest that digitizing PROs could improve patient adherence and that integrating them with physiological data from wearable sensors, such as heart rate, could provide complementary objective biomarkers, thereby enhancing the reliability of pain monitoring [12,13,15-17]. However, there is currently no universally accepted biomarker for pain [12,13,18], and evidence supporting the clinical effectiveness of wearable sensors in palliative cancer care remains scarce. For instance, Sandic et al [16] identified only 6 studies involving wearable technologies in palliative care for patients aged 65 years and older, none of which were conducted in LMICs. Similarly, Cloß et al [19] emphasize the need for further research on the short- and long-term impacts of wearable technologies across different phases of cancer care, while studies from Olmedo-Aguirre et al [6] and Leroux et al [15] point to usability issues and low acceptance of these technologies and stress the importance of additional usability studies.

The NEST (Non-intrusive Devices for Telemedicine) system was developed to address these gaps by designing and piloting a wearable-based telemedicine solution tailored to the needs and constraints of outpatient palliative care in an LMIC setting. This project was established through a collaborative partnership between ESPOL (Escuela Superior Politécnica del Litoral) University and the Society for the Fight Against Cancer (Sociedad de Lucha Contra el Cáncer [SOLCA]) in Ecuador, in cooperation with the Vrije Universiteit Brussel (VUB) in Belgium.

Through a participatory design process with the palliative care team at SOLCA’s hospital, we developed a low-cost, noninvasive smartwatch-based solution paired with a mobile app capable of collecting and transmitting photoplethysmography-derived physiological data and pain-related PROs. This study presents the NEST system and the results of an observational study with the objective of evaluating its implementation feasibility in an LMIC setting and usability among patients with cancer receiving outpatient palliative care within SOLCA’s hospital network. To interpret the findings and assess implementation and scalability potential, we apply the NASSS (Nonadoption, Abandonment, Scale-up, Spread, and Sustainability) framework; a tool developed at the University of Oxford to identify and analyze the sociotechnical factors influencing the long-term adoption of health technologies [20].

This study contributes to the emerging literature by presenting one of the first feasibility and usability studies of wearable technology for pain monitoring in palliative cancer care conducted in an LMIC, addressing a critical gap in evidence from underrepresented settings.

## Methods

**Figure 1.**
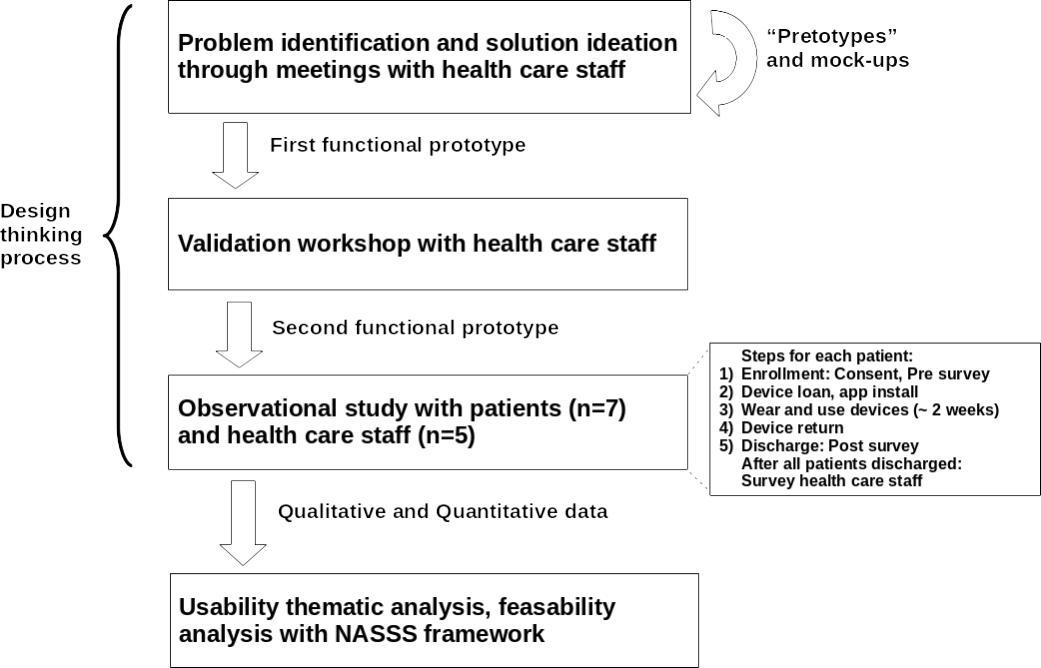
Design and evaluation stages of the NEST (Non-intrusive Devices for Telemedicine) system. NASSS: Nonadoption, Abandonment, Scale-up, Spread, and Sustainability.

Our methodology to design the NEST system and evaluate its usability and feasibility in an LMIC setting comprises a participatory design stage, guided by the principles of Design Thinking, and an observational study for evaluation. We used a thematic analysis for usability and a NASSS framework–guided analysis for feasibility (Figure 1).

### NEST System Design

The NEST system started with the aim to design a sociotechnical solution to support SOLCA’s telemedicine programs. Early on in the start of the project, the telemedicine program of SOLCA’s palliative care unit was identified as the ideal starting point. In total, 17 health care workers (9 doctors, 6 nurses, 1 therapist, and 1 administrative staff) from this unit and 2 specialists from ESPOL (a computer science engineer and a mechatronics engineer) embarked on a participatory design effort using tools from Design Thinking, a human-centered, problem-solving methodology found to be effective at promoting innovation in the health sector [21]. Design Thinking is an iterative process structured around 5 stages—Empathize, Define, Ideate, Prototype, and Test—that emphasizes rapid prototyping and stakeholder participatory design [22]. During the empathy and problem definition phase of the Design Thinking process, we identified pain management as one of the most pressing challenges faced by staff and patients who are ambulatory in SOLCA’s palliative care unit. Specific issues included ensuring appropriate use and dosage of analgesics, as well as consistent completion of the “pain diary,” a customized PRO questionnaire that patients or caregivers were expected to log regularly.

During the ideation and prototyping phases of the design process, a wearable device for logging PROs and measuring digital biomarkers emerged as a promising solution. Early in the design process, insights gathered from meetings with health care staff helped define the essential features of the proposed solution. The wearable device should adopt a smartwatch form factor to remain small and lightweight, minimizing interference with users’ daily activities. At the same time, it should incorporate the largest feasible touchscreen to enhance accessibility, complemented by both virtual and physical buttons. The inclusion of physical buttons was strongly emphasized by clinicians from SOLCA, as their manipulation served as a therapeutic distraction during pain episodes. The device should also offer reasonable water resistance and the capability to continuously measure at least heart rate (other digital biomarkers were a plus but not essential). To ensure usability and redundancy, PRO entry should also be possible via a companion mobile app, which offers a larger display. Additionally, health care staff should have access to a web-based dashboard to visualize patients’ health data in real time.

### NEST System Architecture

**Figure 2.**
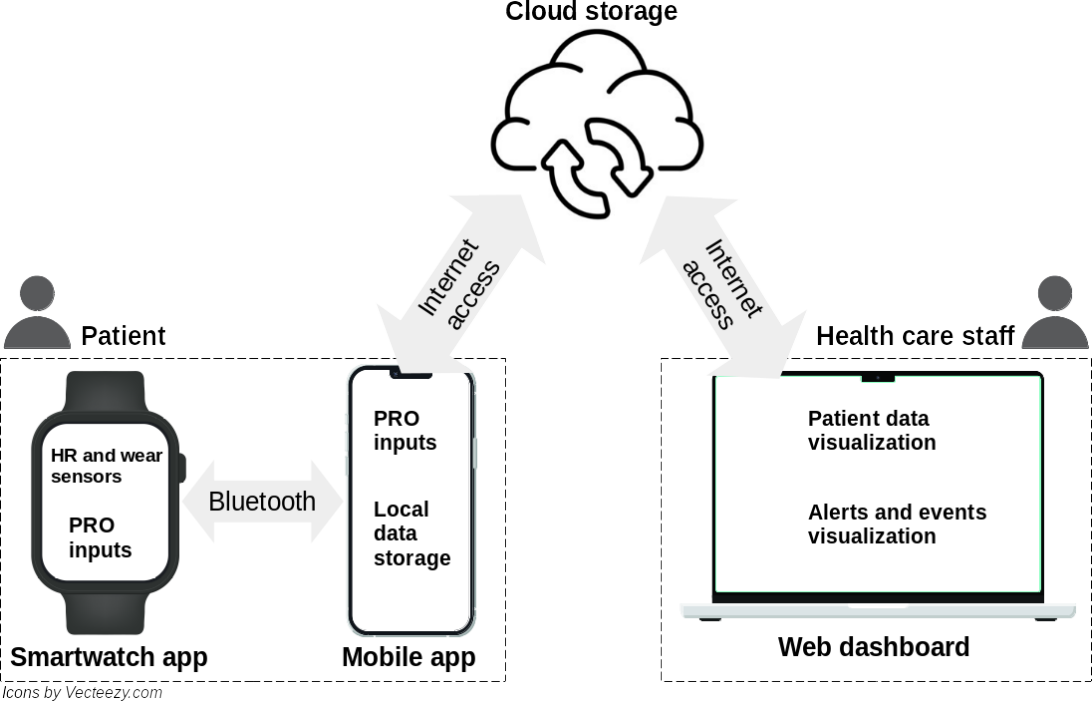
The NEST system consists of 3 components, including a smartwatch, a mobile app, and a web dashboard. HR: heart rate; PRO: patient-reported outcome.

For the last 2 phases of the design process, prototype and test, we developed a functional prototype resembling as much as possible the features collected in the previous phases. This functional prototype is the first version of the NEST system and consists of 3 components including a smartwatch, a mobile app, and a web dashboard. Figure 2 depicts the NEST system architecture and how its components interact with each other and with their respective users.

One of the primary features of the NEST smartwatch is the pain diary, which enables patients to complete a structured pain assessment PRO. This feature, derived and adapted during the initial design process from the original paper–based pain diary used by SOLCA’s staff, consists of 4 questions that prompt users to describe the type of pain they experience, assess whether it limits their daily activities, rate its intensity on a predefined scale, and select the precise pain location using an interactive body map interface. Additionally, users can register an emergency use of their prescribed analgesic, called a rescue, using a designated button function on the watch. The smartwatch also provides real-time heart rate monitoring, displaying the patient’s current heart rate directly on the screen. All registered data, pain diary answers, rescue events, heart rate, and battery level are transmitted to the NEST mobile app via Bluetooth for further processing. Screenshots of the NEST smartwatch, the NEST mobile app, and the NEST web dashboard can be found in Multimedia Appendix 1.

All the features described above were iteratively refined and tested through 2 participatory design workshops with staff from SOLCA’s palliative care unit. To validate the final prototype in a real-world context with patients who are ambulatory, an observational study was conducted. The methodology of this study is detailed in the following section.

### Participants and Protocol

Our study was designed as an observational study for product usability, as our main objective was to evaluate the usability and feasibility of the NEST system within the real day-to-day workflow of SOLCA’s staff.

Participants were recruited by SOLCA’s palliative care team through the home-based care program, which provides medical visits to patients who are unable to travel regularly to the hospital for consultations. Upon initial contact with the patient, at home or at the hospital, a member of the team performed a direct clinical evaluation to assess the patient’s Karnofsky Performance Status (KPS), a widely validated objective measure (ranging from 0 to 100) to assess patient survival in oncology clinical trials [23]. This evaluation, following KPS criteria, was based on observation of the patient’s functional status and a structured clinical interview to determine the patient’s ability to perform daily activities, their level of dependence, and their need for medical assistance. Following the evaluation, if the inclusion and exclusion criteria were met, the clinician informed the patient of the NEST project, its objectives, benefits, and expected commitments. If the patient agreed to participate, recruitment began by obtaining written informed consent directly from the patient, with a family member present to support the process. Inclusion criteria were (1) adult patients with cancer capable of providing informed consent, (2) a KPS above 50, (3) current pain requiring monitoring, and (4) ownership of an Android smartphone (although one could be provided on loan if needed). Exclusion criteria included cognitive impairment, physical limitations preventing smartwatch use (eg, edema or cachexia), lack of family support, residence outside the city, and being younger than 18 years.

The protocol was divided into two phases: (1) hospital-based monitoring for one week and (2) home-based monitoring for one week. During both phases, participants wore the NEST smartwatch continuously and logged their PROs and rescue medication use through the smartwatch or the NEST mobile app. At recruitment, the patient and a family member received basic training on the use of the NEST system by SOLCA staff. Additionally, a small, structured interview was conducted to collect demographic data on the patient. The palliative care team conducted daily check-ins during the hospital phase and remote follow-ups every 2 days during the home phase.

After the conclusion of the hospital and home phase, a final in-person evaluation and structured interview were conducted to collect qualitative feedback on patient user experience. This interview took place at the hospital when the patient returned the wearable device and was administered by a member of the SOLCA staff. All questions were read aloud to the participants. Given the patients’ delicate emotional and physical condition at the conclusion of the study, their responses were not audio-recorded; instead, the interviewer took written notes.

In parallel, qualitative feedback was also obtained from health care staff who had direct contact with participants and the NEST system. This feedback was gathered via a structured survey sent and collected by email at the end of the study.

We designed both questionnaires to accommodate the specific constraints of our palliative care context, where health care staff face high workloads and patients are likely to be in fragile health toward the end of the study. As a result, both interviews and surveys were intentionally kept brief and focused. Several questions were adapted from the mHealth App Usability Questionnaire (MAUQ) developed by Zhou et al [24], which evaluates app usability in health care settings for both patient- and provider-facing stand-alone mobile apps. Modifications were made to account for the real-time data synchronization capabilities of the NEST app and the inclusion of a wearable device, which made the system deviate from a typical stand-alone configuration as defined in the MAUQ.

The full interview and survey scripts are available in Multimedia Appendix 2.

### Devices and Data

The smartwatch models used in the study were the Samsung Galaxy Watch 5 (SM-R910, 44 mm screen) and Galaxy Watch 6 (SM-R940, 44 mm screen), as previous studies have validated the suitability of these smartwatch models for patient home monitoring [25]. These devices were delivered to participants with the NEST WearOS app preinstalled. At recruitment time, the NEST mobile app was installed on each participant’s phone and synchronized with the smartwatch by SOLCA’s staff; a technician from ESPOL was on hand for support during this stage.

The photoplethysmography sensor on the smartwatch was used to obtain heart rate measurements and to assist with wrist detection. The inertial measurement unit on the wearable was used solely to complement wrist detection by identifying motion patterns indicative of active wear. Both heart rate and wrist detection were derived from processed data generated by the device’s proprietary internal algorithms. No raw sensor data were collected during the study. In summary, collected data included the following:

1. Time-stamped pain diary entries and rescue usage reports
2. Time-stamped heart rate measurements
3. Device usage metadata (watch battery level, report source, and device status)
4. Qualitative feedback from participants on usability, comfort, and perceived usefulness
5. Qualitative feedback from health care personnel on usability, impact, integration, and perceived usefulness.

All quantitative data were automatically synced to a secure server accessible to SOLCA’s clinical team through the NEST web-based dashboard.

### Ethical Considerations

The study received ethical approval from the Human Research Ethics Committee of the Universidad Técnica de Manabí on August 12, 2024 (approval code CEISH-UTM-EXT_24-06-21_FXDB). Patient screening and recruitment commenced shortly thereafter and ended on September 15, 2024. Only palliative care unit staff interacted with participants. All participants signed an informed consent form prior to participation and received no compensation for participating. All personally identifiable information was kept as confidential by SOLCA, and the participants’ identities were anonymized using sequential numerical identifiers (User1, User2, etc) in data transmissions and in reports shared with the rest of the project.

## Results

### Overview

Overall, 7 patients from SOLCA’s palliative care unit volunteered and met the inclusion criteria for participation in the study. Around 4 were recruited during outpatient consultations at the hospital and 3 during home-based care visits. Of these, 5 patients used the NEST system for more than 1 week, and 4 patients completed the study by using the system for 2 weeks or more. The study was conducted over a 7-week period in Q3 2024.

Table 1 details the demographic characteristics of the 7 patient participants. In addition to these patients, 5 members of the palliative care unit participated in the study, including 3 medical professionals and 2 nurses, who provided clinical support. Notably, 3 of these health care providers are coauthors of this study. One of the participating physicians also offered technical assistance to the patients, having received prior training on the use of the wearable devices. Detailed disaggregated demographic data of all participants are provided in Multimedia Appendix 3.

**Table 1.** Demographic characteristics of patient participants (N=7).

Characteristics	Value
Age (years), range (median)	35‐77 (47 )
Gender, n (%)	
Man	1 (15)
Woman	6 (85)
Marital status, n (%)	
Single	2 (29)
Married	3 (43)
Divorced	1 (14)
Widowed	1 (14)
Self-reported socioeconomic status, n (%)	
Low-income household and high school diploma	2 (29)
Middle-income household and higher education	5 (71)

User 1 was unable to cooperate with the medical team at the end of the study, and as a result, the closing interview was not conducted, and the smartwatch was not returned. User 5 experienced an extended hospital stay, and the medical team judged that it was best to remove the smartwatch during the closing interview at the end of his hospitalization. User 6 chose to withdraw from the study after 1 day due to her rapidly deteriorating health condition. User 7 encountered an unrecoverable technical failure with her mobile phone after 2 days, which prevented further participation.

### Quantitative Analysis

**Figure 3.**
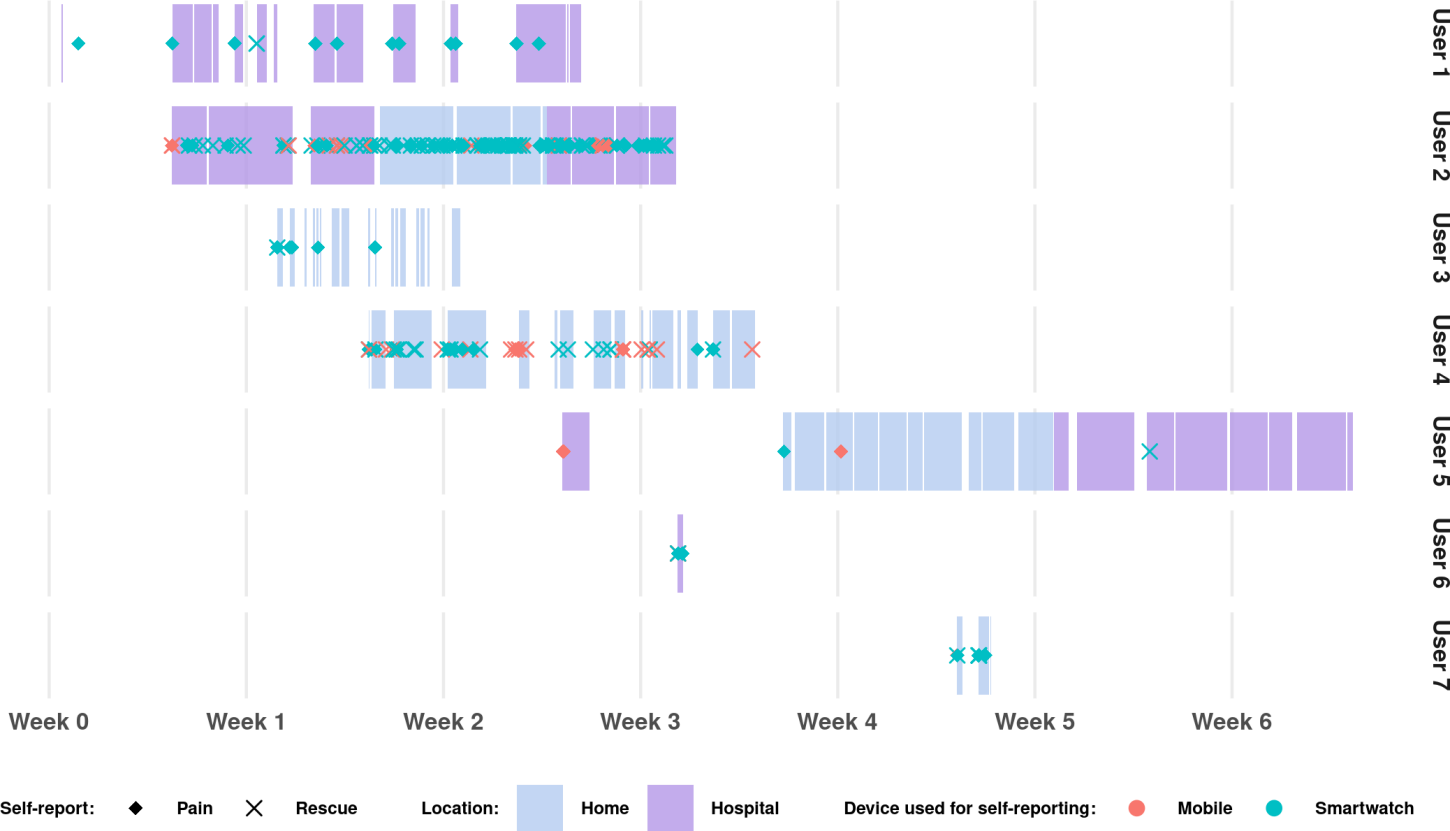
Telemetry pings were used to infer when the NEST (Non-intrusive Devices for Telemedicine) watch was actively worn.

The NEST watch transmits a telemetry ping to the cloud database every 5 minutes while worn on the participant’s wrist. Each ping includes the current heart rate reading and battery level of the device. When combined with participants’ self-reported events (pain and rescue), these pings offer valuable insight into usage patterns of the NEST system by allowing the reconstruction of individual timelines throughout the study. Figure 3 presents a visual timeline of each participant’s interaction with the NEST system (identified as User 1, User 2, etc). Colored bands indicate periods during which participants wore the NEST watch, either at home or in the hospital. Distinct point shapes mark PROs, either self-reported pain or rescue medication events, recorded via the NEST watch or mobile app. Usage data (eg, watch wear time and event reports) were retrieved from the NEST system database, while location information (home or hospital) was derived from anonymized reports provided by SOLCA’s palliative care unit.

**Figure 4.**
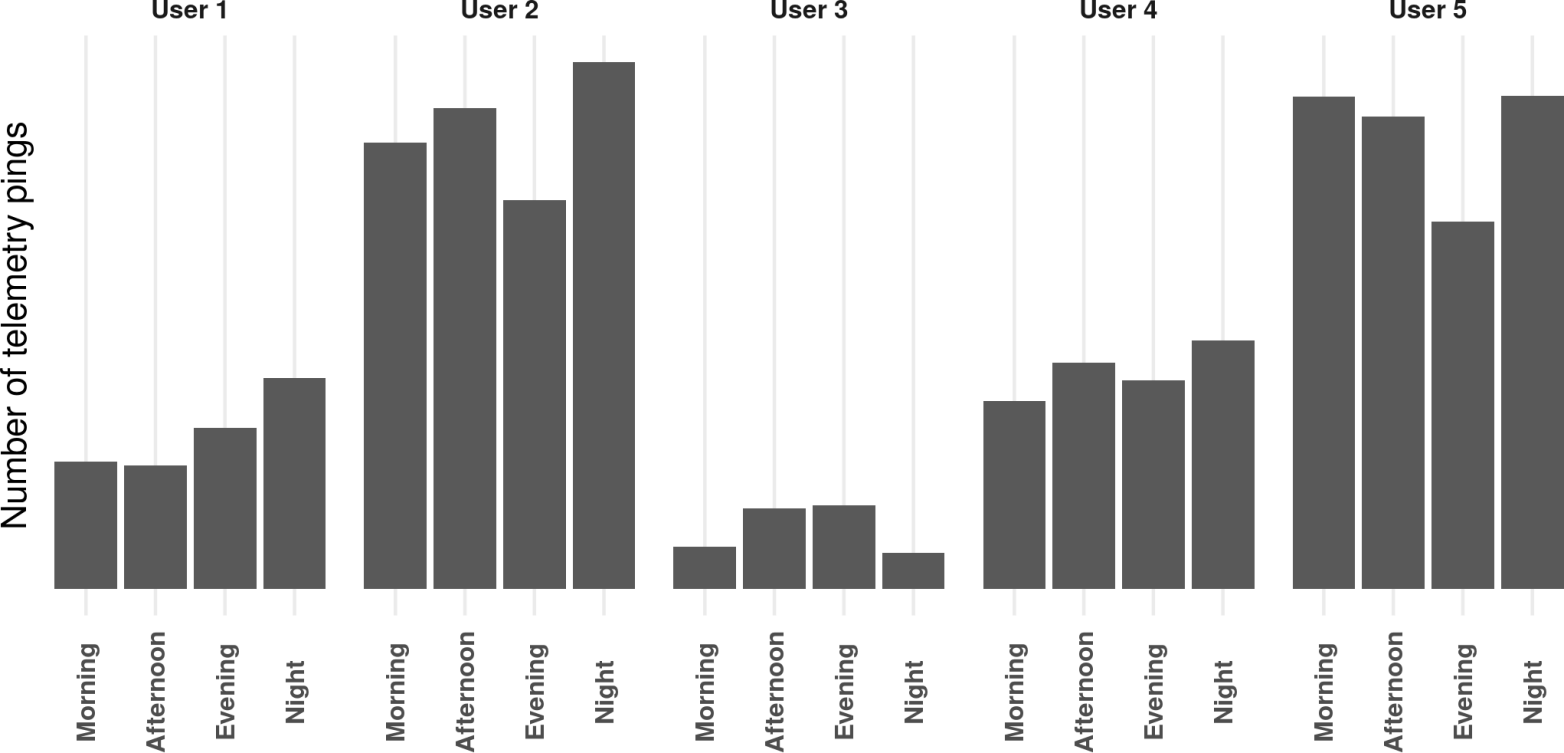
The number of telemetry pings sent to the cloud database is used here to estimate when the NEST (Non-intrusive Devices for Telemedicine) watch was worn with respect to the time of day.

Table 2 summarizes the NEST watch usage for each participant. “PROs” is the total amount of self-reports (pain diary and rescue) per participant, and “Possession” refers to the total number of days participants had the device assigned to them. “Wear time” indicates the total number of hours the watch was actively worn during that period. “Wear rate” represents the percentage of time the device was worn relative to the total possession period. Table 3 breaks down the distribution of self-reports by input method, either the NEST watch or the NEST mobile. Figure 4 presents a breakdown of the number of telemetry pings received per participant across different times of day. This figure reveals that, except for User 3, users mostly used the smartwatch at night during sleeping hours. Users 6 and 7 were omitted from these statistics because they only used the NEST watch for a day or less, precluding the possibility of discerning daily patterns in their data.

**Table 2.** NEST^a^ watch usage per participant.

User	PROs^b^ (n)	Possession (days)	Wear time (hours)	Wear rate (%)
User 1	15	18.4	176	39.8
User 2	193	17.9	396	92.1
User 3	6	6.5	55.9	35.8
User 4	56	13.7	189.8	57.6
User 5	5	28.1	460	68.3

a NEST: Non-intrusive Devices for Telemedicine.

b PRO: patient-reported outcome.

**Table 3.** Distribution of patient-reported outcomes for pain and rescue reports by input method.

Input method	PROs^a^		Total PROs, n (%)
Pain reports (n)	Rescue reports (n)
NEST^b^ mobile	19	31	50 (17)
NEST watch	124	122	246 (83)

a PRO: patient-reported outcome.

b NEST: Non-intrusive Devices for Telemedicine.

Among users 1 through 5, adherence to wearable use (defined as wearing the smartwatch on the wrist) varied, with User 2 showing the highest usage, 92% over 18 days, and User 3 the lowest, 36% over 7 days. In contrast, PRO inputs showed greater variability, ranging from 193 reports by User 2 to only 5 by User 5 across a 28-day period (Table 2). All participants overwhelmingly preferred the NEST watch over NEST mobile for PRO inputs (Table 3), and no statistically significant dependency was found between input method, location, and self-report type.

Previous studies have identified limited battery life and the need for frequent charging as significant barriers to the adoption of wearable devices in health care settings [11,25]. Accordingly, this study included an evaluation of how these factors impacted the usability of the NEST system.

Table 4 presents the battery discharge statistics per participant and the number of charges (for the NEST watch) per participant. Discharge rates observed during the study were consistent with those reported by the device manufacturer (up to 40 hours or ~2.5% per hour), suggesting that the NEST WearOS app and typical usage do not significantly impact battery performance. When considered alongside Figure 4, which indicates lower device usage in the evening and increased usage during the night, this pattern suggests that users commonly charged the watch prior to wearing it to sleep. More detailed battery usage statistics can be found in Multimedia Appendix 4.

**Table 4.** Battery discharge statistics and charging frequency by participant.

User	Charges (n)	Discharge duration (hours), mean (SD)	Discharge rate per hour (%), mean (SD)
User 1	6	43.1 (15.2)	1.8 (0.2)
User 2	10	39.6 (17.8)	1.7 (0.3)
User 3	5	22.2 (10.6)	2.7 (0.6)
User 4	7	34.9 (10.5)	2.7 (0.5)
User 5	14	33.2 (14)	2.6 (1.0)

### Qualitative Results

At the conclusion of the study, semistructured interviews were conducted with the 7 participating patients to evaluate their overall experience with the NEST system. These interviews, consisting of 8 open-ended questions on usability, were administered by health care staff from SOLCA and took place upon the formal return of the smartwatch. Additionally, qualitative feedback was collected from the 5 members of SOLCA’s palliative care team who participated in this study in clinical and technical roles.

### Patients

Of the 7 participants, 6 completed the full feedback interview. One participant (User 1) did not return the device, and thus no responses were obtained despite follow-up attempts.

### Usability and Ease of Use

Around 6 of the 7 participants reported that both the wearable device and mobile app were easy to use, indicating that the interface was intuitive and required minimal adaptation time. Two participants adapted within days, while another reported immediate ease of use. Only one participant mentioned a specific difficulty related to uncertainty about how to charge the smartwatch.

*It took me 3 days to adapt to the smartwatch and the app*…

[User 3]

*It took me a week to adapt to the smartwatch*…

[User 4]

### Technical Functionality

User 6 reported that the watch disconnected from the mobile app after a few days and therefore stopped using the device. User 2 noted minor, unspecified technical problems, whereas the rest reported smooth functionality throughout the study.

### Pain Management Support

In total, 5 of the 7 participants perceived the device as beneficial for managing their pain. One participant emphasized the usefulness of being able to track medication frequency through the app:

Thanks to the device, I could easily see how many rescues I used throughout the day. This made it easier to track my pain and the number of interventions needed.

[User 2]

### Suggestions for Improvement

Users recommended several improvements. One participant suggested making the rescue logging function more prominent within the app interface and providing sound alerts if necessary.

Another user expressed interest in a voice-enabled option for enhanced accessibility.

*It would be nice if the smartwatch could talk…*

[User 3]

There was also a suggestion for a more compact device, as one user declined to use the smartwatch outside the study context due to its size. Also, User 6 suggested adding an alert button to the device:

I found the system mildly useful…it would have been beneficial if the smartwatch was linked to the nurses’ station… [for alerts]

[User 6]

### Daily Life Integration and Future Use

In 4 of the 7 participants expressed a willingness to continue using the wearable system beyond the research setting. Reasons included its practicality, its usefulness for tracking pain and activity levels, and the added sense of monitoring and safety. One participant specifically noted the value of step and heart rate tracking for daily well-being. In contrast, User 2 was reluctant to use the device regularly due to its size.

### Health Care Staff

A questionnaire with 14 questions on user experience, impact on pain management, integration into the hospital environment, suggestions, and ethical considerations was distributed to the 5 members of SOLCA’s palliative care team who had direct contact with participating patients. Table 5 outlines the roles within the project of the health care personnel involved.

**Table 5.** Demographic characteristics of palliative care unit staff involved in patient-facing activities during the study.

Participant ID	Gender	Role	Experience in palliative care (years)
D1	Woman	Project supervisor	3
D2	Man	Medical support	1
D3	Man	Medical and technical support	3
N1	Woman	Nursing support	3
N2	Woman	Nursing support	2

### User Experience

Overall, staff reported that patients found the NEST watch and mobile app moderately easy to use. While most participants adapted to the system quickly, initial challenges were observed with setup and charging. Three staff members emphasized that the training provided to patients was insufficient in some cases, especially regarding device configuration and battery management. Suggestions included improving initial training or incorporating hands-on practice sessions prior to deployment. The project supervisor noted:

*It was a good experience, [the system] provided monitoring to selected patients and they were attentive to its use as a good tool that gave them security.*

[D1]

However, one of the attending nurses remarked:

I heard a lot of comments about how the main problem was charging the watch.

[NI]

From the staff perspective, the web-based monitoring dashboard was consistently described as clear and easy to use. The user interface enabled timely access to pain reports and physiological data, although occasional connectivity or synchronization issues were noted.

### Impact on Pain Management and Clinical Workflow

All clinicians observed positive changes in patient behavior related to pain reporting. Compared to paper-based diaries, the digital system allowed more consistent and immediate self-reporting, which was perceived to empower patients and support open discussions about pain management. The attending nurses remarked:

*It was gratifying to see how some patients felt empowered by being able to monitor their own pain …*

[NI]

[the system] … facilitated more accurate and timely decisions, especially in the administration of painkillers.

[N2]

However, the project supervisor was more cautious, suggesting the influence of the Hawthorne effect, where individuals modify their behavior because they are aware of being observed [19], on the more consistent use of the digital system. Moreover, 2 respondents cautioned that not all participants used the device continuously, which limited its influence on clinical decision-making.

Regarding physiological monitoring, views were mixed. Some professionals saw the device’s continuous tracking as a promising complement to traditional methods, while others felt that its clinical value was limited without further optimization or integration with other systems. About the clinical usefulness of the data, the doctors remarked:

I don’t think [the information collected by the device] is clinically useful, but it has allowed tracking without the need for direct contact, which sometimes takes a little longer.

[D1]

It is a useful tool, but I don't think it replaces traditional methods.

[D2]

It is no more useful than performing a direct interview with the patient.

[D3]

### Integration Into Hospital Environment

Staff were optimistic about the compatibility of the NEST system with existing palliative care workflows. While full integration with electronic health records was not implemented during the study, all respondents agreed that the system could be linked effectively with telemedicine platforms or other digital tools to enhance multidisciplinary coordination.

The long-term viability of the device was considered plausible but dependent on certain conditions such as improved patient onboarding, dedicated technical support, and clear allocation of staff resources for monitoring.

### Suggestions for Improvement

Common suggestions for improvement included adding medication reminders, improving battery life notifications, and incorporating features for broader tracking of symptoms (eg, anxiety or physical activity). All respondents emphasized the need for ongoing technical support, including troubleshooting, software updates, and user training, particularly given the variable levels of digital literacy among patients.

## Discussion

### Principal Findings

This study addresses a critical gap in the current literature by exploring the feasibility and usability of wearable technology for pain monitoring in palliative cancer care within an LMIC setting. To our knowledge, it is among the first studies to explore this technology in such a context. For usability, limited battery life emerged as the primary usability challenge, as reported in qualitative feedback from both patients and health care staff and as reported in previous similar studies [11,25]. Nevertheless, usage logs indicated that 5 of 7 patients learned to routinely charge the smartwatch overnight and wore it during sleep. Although the small screen was noted as a barrier, all users still preferred the smartwatch over the smartphone for reporting PROs. Together with generally positive qualitative feedback, these findings suggest that typical usability barriers associated with wearable technologies can be successfully mitigated in this context.

As for the feasibility of implementation, we used the NASSS framework as a lens to identify two key issues: (1) the complexities and unknowns to manage toward scaling up and (2) long-term adoption of our sociotechnical solution. Our main insight is that, in our LMIC context, most of the complexity was found in the dynamics of the health condition while the technology shows clear promising signs of having value to patients and health care staff. However, a larger study is necessary to address the complexities inherent in palliative cancer care and to explore three key uncertainties: (1) how to enhance the system’s value proposition by integrating it into the workflow of a palliative care unit, (2) what the actual cost–benefit balance of the technology is for health care organizations, and (3) how to manage third-party technological dependencies within an evolving and often ambiguous regulatory landscape for biomedical devices. These findings add to the emerging body of literature on digital health interventions in palliative care by uncovering the potential and pitfalls of wearable solutions in resource-constrained environments.

### NASSS Framework Analysis

**Figure 5.**
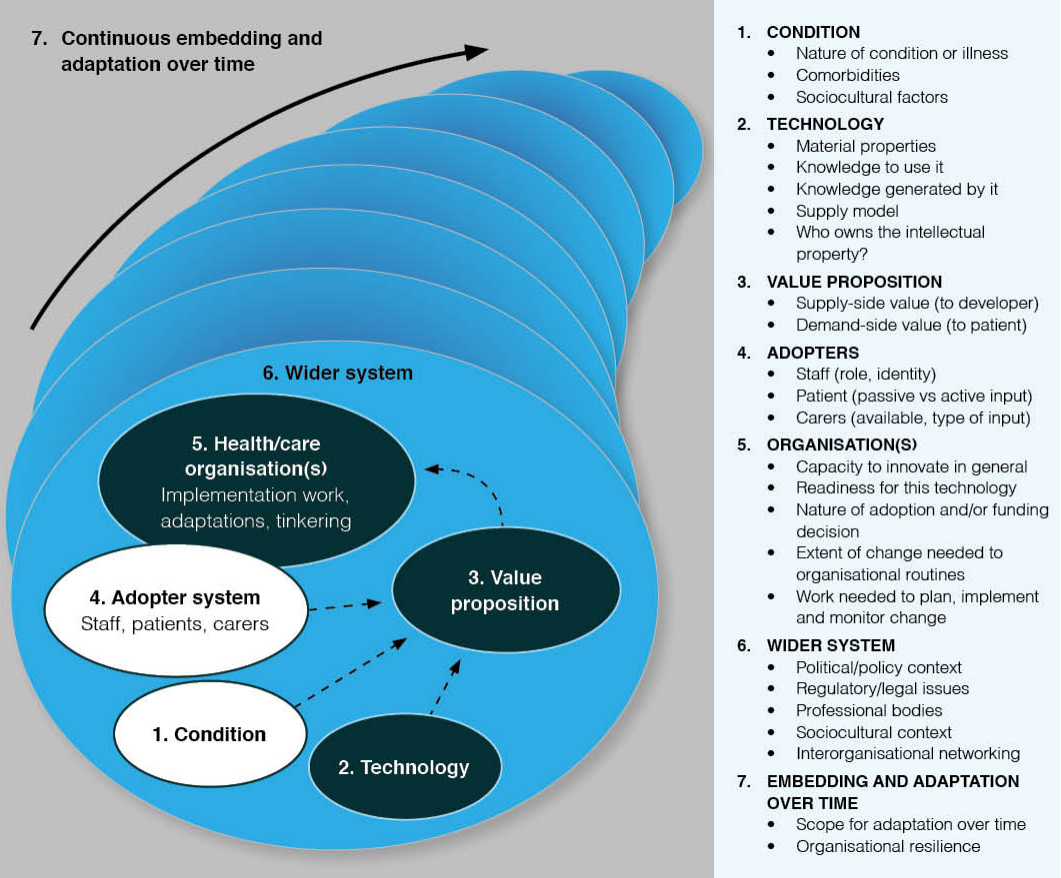
The NASSS (nonadoption, abandonment, scale-up, spread, and sustainability) framework helps to inform the design of new eHealth technologies (reproduced from Greenhalgh et al [26], which is published under Creative Commons Attribution 4.0 International License [CCBY reference citation #^27^).

The NASSS framework, developed by Greenhalgh et al [20] in 2017 at the University of Oxford, provides a comprehensive approach for evaluating the implementation of health technologies across sociotechnical contexts. The framework consists of seven domains: (1) the health condition, (2) the technology, (3) the value proposition, (4) the adopter system, (5) the organization, (6) the wider context, and (7) the interaction and adaptation over time (refer to Figure 5 for more details). Each domain can be categorized as simple (predictable, few components), complicated (multiple interacting elements), or complex (dynamic, uncertain, and difficult to disaggregate). When multiple domains are classified as complex, implementation and long-term adoption tend to be significantly hindered [20].

In the case of the NEST system, the results of the observational study can be examined ex-post through the NASSS framework to identify current strengths, challenges, and areas requiring further development [20,26,28]. To reflect the exploratory nature of this study, we introduce a fourth category, to be determined, to denote domains where insufficient evidence prevents definitive classification. A detailed discussion and classification of each domain is provided below.

### Condition

The condition addressed in this study is cancer, at any stage, requiring palliative care. Palliative care delivery is inherently individualized, shaped not only by the patient’s clinical needs but also by the needs of companions and caregivers [4]. The complexity of this condition is heightened by the frequent presence of comorbidities and the dynamic nature of symptoms, particularly in the outpatient context. In our context, sociocultural factors further complicate care. Palliative care is often stigmatized or misunderstood, and its objectives may be dismissed or resisted by patients’ families and social networks. These challenges were reflected in this study through recruitment difficulties leading to limited sample size, as well as issues with participant retention and study protocol adherence. This domain is therefore classified as complex.

### Technology

The NEST system comprises 3 interconnected components, including the NEST watch, the NEST mobile app (for Android smartphones), and the NEST web dashboard. These components must communicate with each other in soft real-time to ensure the timely and accurate exchange of health data between patients and health care providers. In general, qualitative feedback from all stakeholders was largely positive. Notably, patients had a clear preference for using the smartwatch interface to submit self-reports, despite initial concerns about the small screen size. This preference remained consistent in both hospital and home settings.

However, several challenges were identified during the study (refer to Textbox 1), and these technical limitations suggest that this domain is best classified as complicated.

Textbox 1. **Technology domain challenges.**

- NEST (Non-intrusive Devices for Telemedicine) watch: Although the customized software did not significantly reduce battery life, remaining close to the manufacturer’s specified discharge duration, users typically needed to recharge the device every 2 days or less.
- NEST mobile: The diversity of devices within the Android ecosystem posed challenges for app setup and contributed to variability in performance and reliability across users.

### Value Proposition

The value proposition of the NEST system can be considered across the 3 primary stakeholder groups, namely health care staff, patients, and institution (refer to Textbox 2 ).

Textbox 2. **Value proposition for all stakeholders.**

- Health care staff: Perceived value among health care professionals ranged from moderate to high. Poststudy feedback was generally positive, though some reservations were made, particularly regarding the limited use of the dashboard. The dashboard represented an additional interface to manage during their already demanding workflows. This underusage may reflect workflow integration challenges or limited perceived utility, indicating a potential barrier to realizing full value in clinical practice.
- Patients: Both qualitative feedback and quantitative usage data suggest that patients derived clear value from using the NEST (Non-intrusive Devices for Telemedicine) system. Participants engaged consistently with the wearable and mobile app, indicating acceptability and perceived usefulness. However, the extent and sustainability of this value require further investigation through long-term studies and larger samples.
- Institution (SOLCA [Sociedad de Lucha Contra el Cáncer]): From an institutional perspective, the financial burden of implementing and scaling the system, including the cost of smartwatches, backend infrastructure, and support, would likely fall on the health care provider rather than the patient. Thus, the long-term institutional value of NEST hinges on a more comprehensive evaluation of its impact on clinical outcomes, workflow efficiency, and cost-effectiveness.

Given these uncertainties, particularly around integration into clinical workflows and institutional cost-benefit, this domain is best classified as to be determined.

### Adopters

The primary adopters of the NEST system are health care staff within the palliative care unit and their patients. The early involvement of health care staff in the design process translated later into a critical but positive attitude toward the system and its perceived usefulness. This is key to ensuring future adoption, and it is consistent with empirical findings on similar palliative care sociotechnical solutions [7,29].

For health care staff, the NEST system supplements rather than replaces existing telemedicine tools, offering additional value without significant disruption to established workflows. However, some reservations were noted regarding the consistent use of the dashboard and the need for additional training, suggesting that further refinement or integration with existing clinical systems may be needed to support regular use.

As for patients, study data indicate that patients were generally willing to engage with technology, particularly when encouraged or instructed by staff. Given the variation in patient readiness and the need to address integration issues for staff, this domain is best classified as complicated.

### Organization

SOLCA, the hosting institution for the study, demonstrates openness to innovation and a willingness to explore new technologies for improving patient care. However, as a semiprivate organization reliant on government funding and operating under budgetary constraints, large-scale adoption of the NEST system would require careful financial consideration. Future adoption will depend on lowering investment costs (cheaper wearables, less reliance on technical support, etc) and convincing key stakeholders of the technology’s value, and for this, a more extensive study is needed. This domain is best classified as to be determined.

### Wider System

External risks beyond SOLCA’s control, such as potential government funding cuts, pose a threat to sustained adoption. Although no issues were noted regarding user or data safety, the regulatory landscape for medical devices in Ecuador remains unclear and potentially contradictory. Given that these uncertainties will have to be managed, this domain is best classified as complicated.

### Embedding and Adaptation Over Time

The growing need for palliative care suggests an expanding potential user base, strengthening the long-term value proposition of the NEST system. However, the system’s current reliance on third-party commercial platforms (eg, Samsung wearables and Google’s WearOS) introduces significant limitations. These platforms are typically high-end consumer products, subject to proprietary constraints, limited researcher access to raw sensor data, and opaque data processing algorithms [13,30,31]. Furthermore, future updates to these platforms may inadvertently disrupt existing deployments, undermining system stability and continuity of research.

This reliance is particularly problematic in the context of optimizing digital biomarkers for LMICs. For instance, photoplethysmography sensors embedded in commercial wearables are often calibrated primarily for lighter skin tones [32,33], reducing accuracy for diverse populations. Additionally, the development of novel digital biomarkers better suited for pain management remains constrained by restricted data access.

This complexity could be managed by pursuing a custom-made low-cost hardware solution, but this in turn requires navigating the regulatory environment, which remains insufficiently defined and may pose challenges to sustained adoption. These factors highlight the need for further research. Given these uncertainties, this domain is best classified as to be determined.

### Limitations

The main limitation of this study is the sample size. It is therefore underpowered for a more thorough quantitative analysis and unbalanced in several demographic variables (eg, gender, socioeconomic status, and education). Also, positive qualitative responses may be influenced by novelty and Hawthorne effects [19], wherein participants alter their behavior or report more favorable experiences simply due to heightened attention during the study period. Palliative care in patients with cancer is a dynamic and complex environment, especially in an LMIC setting, where patients are extremely vulnerable and health care staff are normally underpaid, overwhelmed, and work with limited resources. Any study in this context will face challenges regarding retention, engagement, and data quality. Despite these constraints, prior research suggests that usability testing with as few as 5 participants can reveal most usability issues [34,35]. Moreover, Greenhalgh et al [20,26] have advocated for applying the NASSS framework early in the development of sociotechnical health solutions to ensure alignment with the clinical context and inform the design of future, larger-scale evaluations.

### Conclusion

Our findings suggest that the commonly reported usability hurdles of a smartwatch-based sociotechnical health solution may be surmountable given fluid communication between stakeholders during all stages of design and deployment. While our results should not be broadly generalized to all LMICs due to regional variability, the commonly held concerns regarding feasibility in LMICs, such as unreliable internet access and the high cost of internet-capable devices [36-38] were not observed in our study. Instead, the primary threats to feasibility in our context seem to lie rather in the highly complex and dynamic environment of palliative cancer care, regulatory ambiguity regarding the use of medical devices, and the workload burden on health care staff. To navigate this complexity, future scale-up efforts could benefit from applying complexity assessment tools developed by Greenhalgh et al [26] as part of the NASSS framework extension, which supports the implementation of sociotechnical health care systems, such as NEST. These next steps will be critical to ensuring the system’s robustness, scalability, and sustainability in the context of palliative care in low-resource settings.

## Supplementary material

10.2196/78098 Multimedia Appendix 1 Screenshots of NEST (Non-intrusive Devices for Telemedicine) devices.

10.2196/78098 Multimedia Appendix 2 Qualitative data collection scripts.

10.2196/78098 Multimedia Appendix 3 Demographic characteristics of patients receiving palliative care who participated in our study.

10.2196/78098 Multimedia Appendix 4 Detailed battery usage.

## Abbreviations

ESPOL: Escuela Superior Politécnica del Litoral
KPS: Karnofsky Performance Status
LMIC: low- and middle-income country
MAUQ: mHealth App Usability Questionnaire
NASSS: Nonadoption, Abandonment, Scale-up, Spread, and Sustainability
NEST: Non-intrusive Devices for Telemedicine
PRO: patient-reported outcome
SOLCA: Sociedad de Lucha contra el Cáncer
VUB: Vrije Universiteit Brussel
