# Supplementary material for: Evaluating a Wearable-Based Pain Monitoring System in Palliative Cancer Care: Usability and Feasibility Study
Source: JMIR Form Res. 2026 Feb 6;10:e78098. doi: 10.2196/78098 (PMC12880589; doi:10.2196/78098)
Supplement: Multimedia Appendix 1 [file formative-v10-e78098-s001.docx]

Screenshots of NEST devices


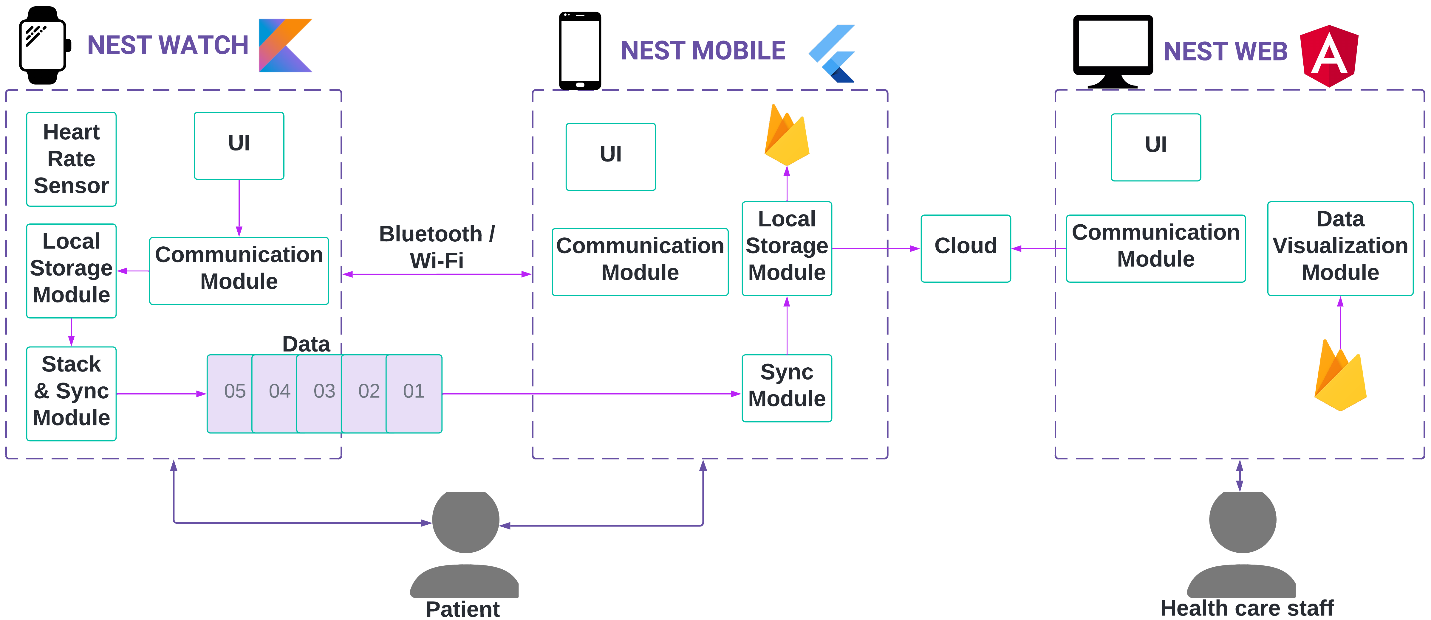


Figure 1. The NEST system consists of three components: a smartwatch, a mobile app, and a web dashboard.


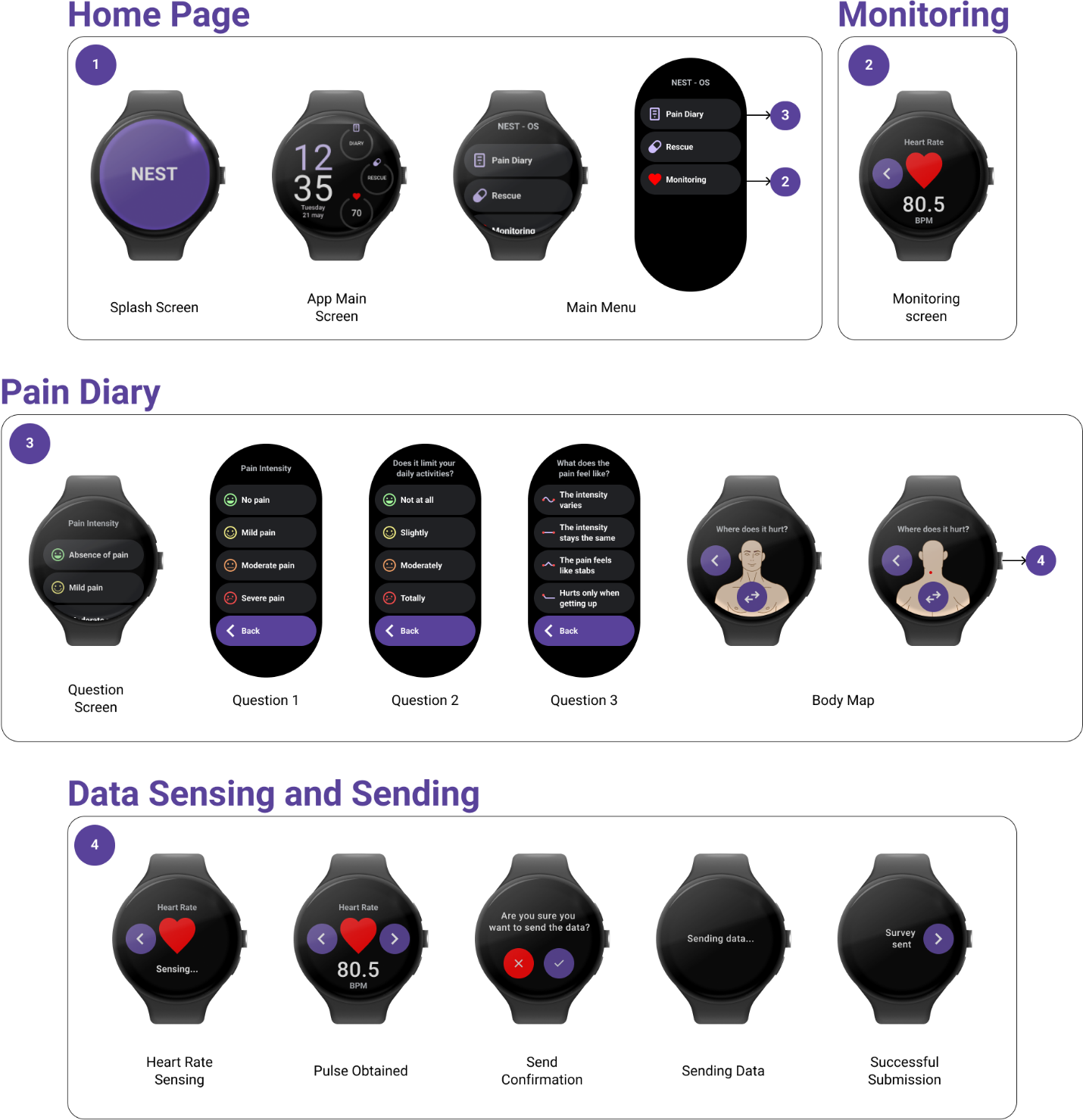


Figure 2. A translated version of an excerpt out of the instruction’s booklet presented to patients and staff on the use of the NEST watch shows the main screen flows.


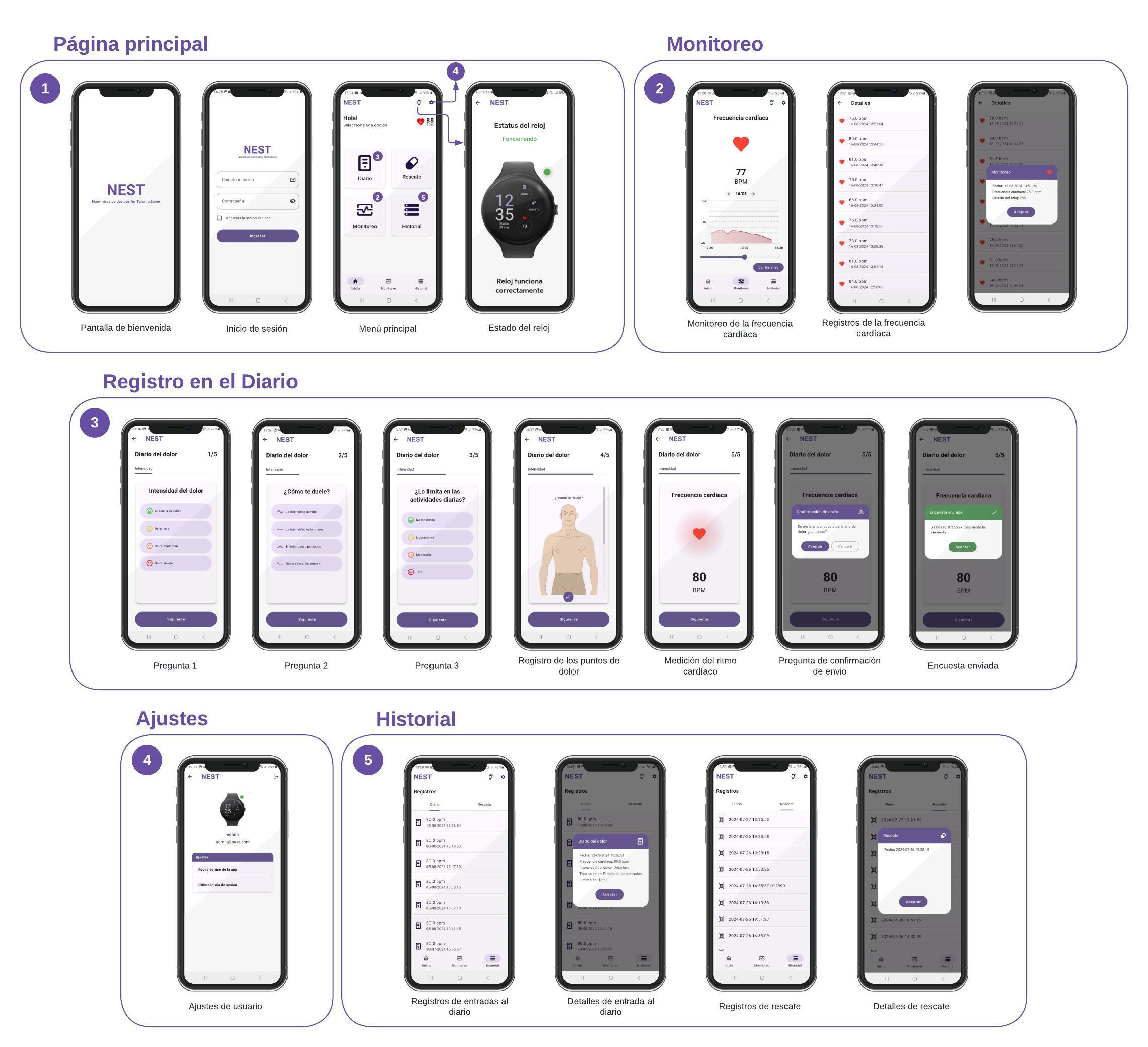


Figure 3. GUI flow in Spanish of the NEST mobile application.


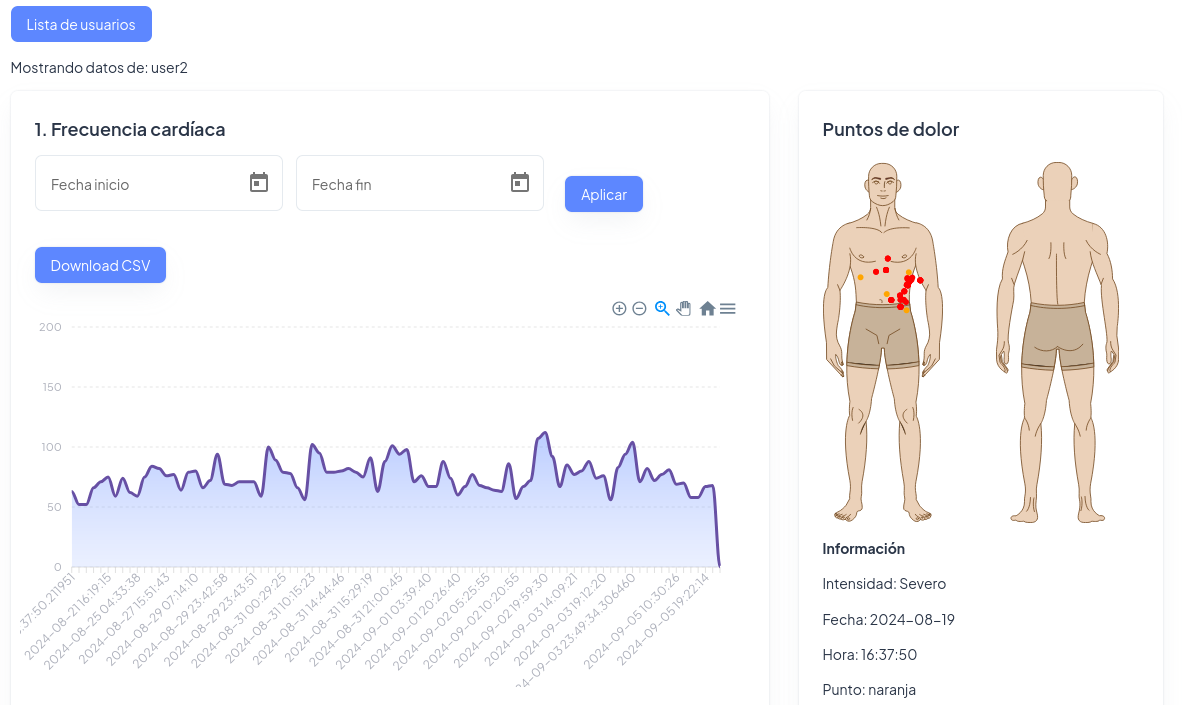


Figure 4. A screenshot of the NEST web dashboard shows how the PRO and heart rate data is visualized by healthcare staff.
