# Supplementary material for: Evaluating a Wearable-Based Pain Monitoring System in Palliative Cancer Care: Usability and Feasibility Study
Source: JMIR Form Res. 2026 Feb 6;10:e78098. doi: 10.2196/78098 (PMC12880589; doi:10.2196/78098)
Supplement: Multimedia Appendix 2 [file formative-v10-e78098-s002.docx]

Scripts of all qualitative data collection

Contents

[Initial data collection from participants 1](#_Toc214595577)

[Final data collection from participants 3](#_Toc214595578)

[Final data collection from SOLCA’s staff 4](#_Toc214595579)

# Initial data collection from participants

The text below is a translated version of the script given to SOLCA’s staff to collect initial data from participants.

*It is completely voluntary to answer all questions. The information in* ***red*** *is confidential and for SOLCA internal use only, it should not be reported to researchers outside SOLCA. The information in* ***green*** *is essential and of high importance for the study.*

***1. Personal Data and Contact***

- ***Full name:***
- ***Age:***
- ***Gender:***
- ***Identification number (card/passport):***
- ***Phone number:***
- ***Email:***
- ***Residence address:***

***2. Marital Status and Family Situation***

- ***Marital status (single, married, etc.):***
- ***Number of children (if applicable):***
- ***Do you live alone or with someone?***
- ***How many people depend on you financially?***

***3. Educational Level and Occupation***

- ***Educational level achieved (primary, secondary, higher, etc.):***
- ***Current occupation:***
- ***Work/study place:***
- ***If you don't work, what is your main source of income?***

***4. Basic Medical History***

- ***Have you been diagnosed with any chronic illness? (Specify):***
- ***Are you currently undergoing any medical treatment?***
- ***Have you been hospitalized in the last year?***

***5. Economic Situation***

- ***What is your main source of income?***
- ***Monthly income range:***
- ***Do you have health insurance? (Yes/No, Specify type):***

***6. Study Specific Conditions***

- ***Do you have regular access to a mobile device and internet connection?***
- ***How comfortable are you with technology in general?***
- ***Have you ever used smart watches?***
  - ***If the answer is “Yes,” how often do you use them?***
  - ***What do you like and dislike about these devices?***
- ***Have you ever used telemedicine devices at home?***
- ***What expectations do you have of a device like the one you are going to use now?***

# Final data collection from participants

The text below is a translated version of the script given to SOLCA’s staff to collect data from participants after finishing the study.

*It is completely voluntary to answer all questions. Questions should be open-ended and allow the participant to report or comment as much as they can. If you consider it necessary to record the interview to facilitate note-taking, it is important to obtain the patient's consent. The recording and notes of the responses must be treated as confidential.*

*1. How easy was it for you to use the NEST Wearable device and mobile app?*

*This question seeks to evaluate the overall usability of the device.*

*2. Were there any parts of the process that you found confusing or difficult to understand?*

*This helps identify specific areas where users encountered difficulties.*

*3. How would you describe your experience recording your pain level using the device?*

*This question focuses on the primary functionality of the device in the context of the study.*

*4. Did you feel the device and app were helpful in managing your pain?*

*The aim is to evaluate the perceived usefulness of the device in personal care.*

*5. Did you experience any technical issues while using the device or app?*

*This allows us to identify technical failures that could affect usability.*

*6. How long did it take you to adapt to using the device and the app*

*This question measures the learning curve associated with using the device.*

*7. Are there any features you would like to add or improve on the device or app?*

*Provides valuable information for the development of future versions of the product.*

*8. Will you use the device in your daily life outside of the studio environment? Why yes or why not?*

*This assesses acceptance and willingness to continue using the device outside the controlled context of the study. If the patient cites the cost of the device as a reason for not using the device, ask if they would use it if the device were loaned or donated.*

# Final data collection from SOLCA’s staff

The text below is a translated version of the questions sent to SOLCA’s staff via email. They were sent only to staff who had contact with participants.

*1. User Experience*

*• How easy or difficult did patients find it to use the NEST Wearable and its mobile app during the study?*

*• How easy or difficult did they find it to use the web dashboard to monitor participants during the study?*

*• Do you feel the training provided to patients was sufficient for them to understand how to use the device?*

*• How would you describe your own experience with the device during your interactions with patients?*

*• Did you encounter any technical problems (connectivity, measurement accuracy, etc.) while using the device or the app?*

*2. Impact on Patient Care*

*• Have you observed any changes in how patients manage and report their pain when using the device compared to traditional methods (such as a paper pain diary)?*

*• Do you believe the device facilitates better monitoring of physiological parameters in cancer patients?*

*• Has the NEST Wearable provided useful information that has improved the quality of clinical decisions in the treatment of patients' pain?*

*3. Integration into the Hospital Environment*

*• How do you perceive the compatibility of the NEST Wearable with other care systems currently used in the palliative care unit?*

*• How viable do you consider the long-term implementation of this type of device in your daily practice?*

*4. Suggestions and Improvements*

*• What additional features do you think could improve the functionality of the device or the application?*

*• Do you consider that ongoing technical support would be necessary to ensure its proper use? If so, what type?*

*5. Ethical and Privacy Considerations*

*• How did you handle patient privacy and data protection when using the NEST Wearable? (This is important considering Ecuador's Organic Law on the Protection of Personal Data).*

*• Did patients express any concerns regarding the collection and handling of their personal data?*

*These questions are designed to gather detailed information about the usability of the device, any obstacles you may have encountered, and how its use could be improved in the future.*
