# Supplementary material for: Evaluating a Wearable-Based Pain Monitoring System in Palliative Cancer Care: Usability and Feasibility Study
Source: JMIR Form Res. 2026 Feb 6;10:e78098. doi: 10.2196/78098 (PMC12880589; doi:10.2196/78098)
Supplement: Multimedia Appendix 3 [file formative-v10-e78098-s003.docx]

## Demographic characteristics of palliative care patients who participated in our study

Table 1. Demographic characteristics

| **Participant identifier** | **Gender** | **Age, years** | **Marital status** | **Children** | **Who do you live with?** | **Self-identified socioeconomic status** | **Education level** | **Income source** |
| --- | --- | --- | --- | --- | --- | --- | --- | --- |
| user1 | Woman | 35 | Single | 2 | Partner | Low-income household | High school | Pension |
| user2 | Woman | 50 | Married | 0 | Partner | Middle-income household | University | Pension |
| user3 | Woman | 64 | Married | 2 | Family | Middle-income household | University | Family |
| user4 | Woman | 47 | Divorced | 1 | Daughter | Middle-income household | University | Family |
| user5 | Man | 77 | Widow | 5 | Family | Middle-income household | University | Pension |
| user6 | Woman | 28 | Single | 3 | Family | Low-income household | High school | Family |
| user7 | Woman | 46 | Married | 2 | Alone | Middle-income household | University | Family |
