# Supplementary material for: Evaluating a Wearable-Based Pain Monitoring System in Palliative Cancer Care: Usability and Feasibility Study
Source: JMIR Form Res. 2026 Feb 6;10:e78098. doi: 10.2196/78098 (PMC12880589; doi:10.2196/78098)
Supplement: Multimedia Appendix 4 [file formative-v10-e78098-s004.docx]

# Detailed battery usage


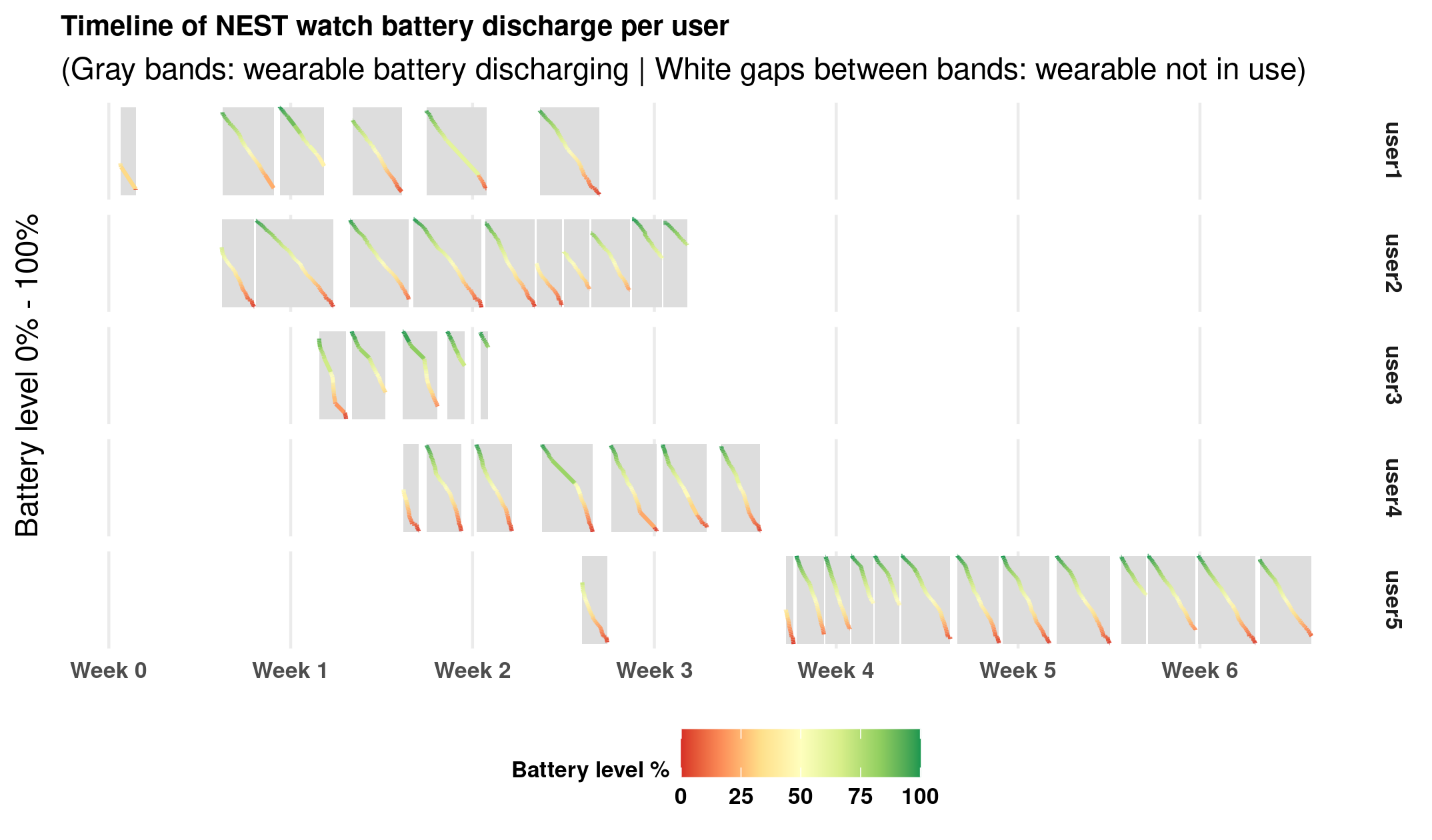


Figure 1. Battery level data, recorded at five-minute intervals, were analyzed to estimate the discharge rate of the NEST watch during use, providing a comprehensive timeline of charging and discharging periods. The white gaps between the gray bands are periods when the smartwatch is either charging or not in use by the participant.


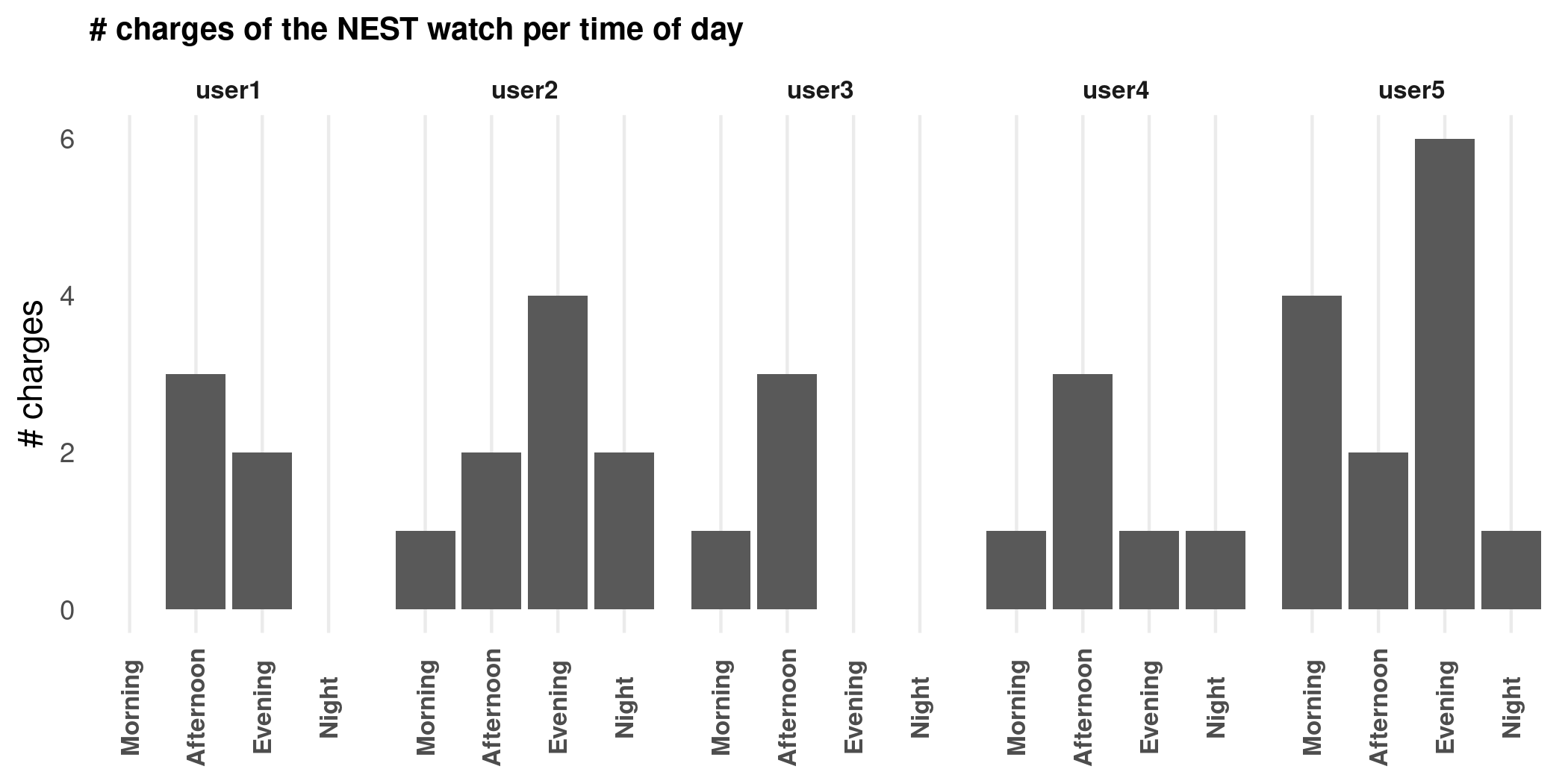


Figure 2. Jumps in the battery level telemetry are used here to estimate when the NEST watch was being charged with respect to the time of day. Most charges are in the afternoon or in the evening.

Figure 1 highlights the periods during which the NEST watch was discharging (indicated by gray bands). The white gaps between the bands indicate the periods where the smartwatch was either charging or not in use by the participant. The graph shows that most users, most of the time, waited for the smartwatch to completely discharge before charging the device. Figure 2 presents the distribution of charging events by time of day, showing that participants frequently charged the NEST watch in the afternoon and evening.
